# Supplementary material for: Oral Microbial Translocation Genes in Gastrointestinal Cancers: Insights from Metagenomic Analysis
Source: Microorganisms. 2024 Oct 18;12(10):2086. doi: 10.3390/microorganisms12102086 (PMC11510655; doi:10.3390/microorganisms12102086)
Supplement: Supplementary file 1 [file microorganisms-12-02086-s001.zip › SuppFigures_241010.pdf]

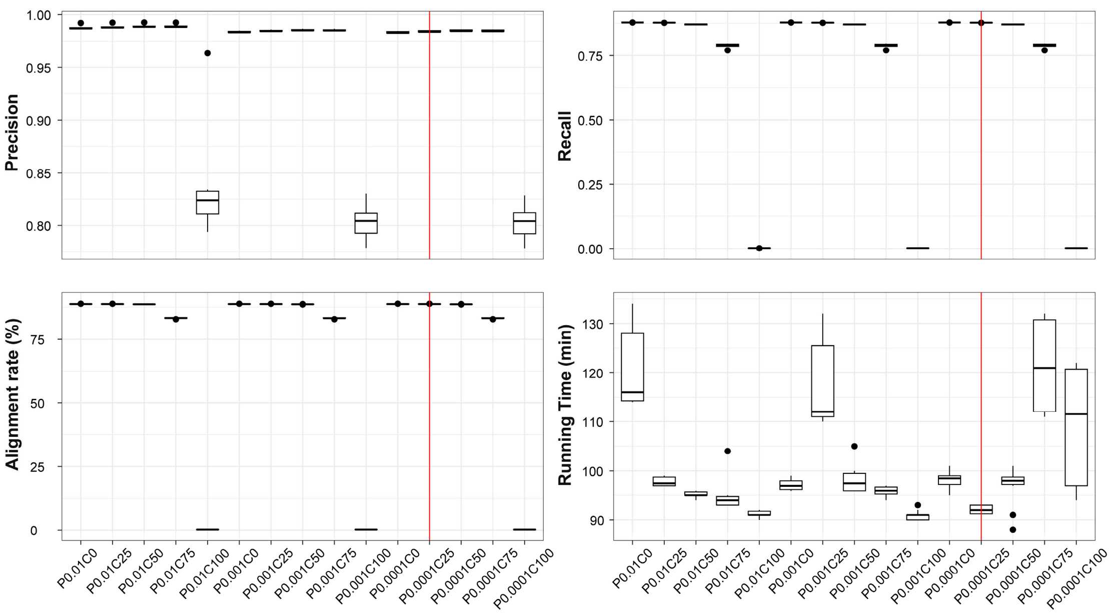

Figure S1. Robustness test of HUMAnN3 parameters based on simulated data. The pre-screen-threshold (P) and nucleotide-subject-coverage-threshold (C) parameters were set at three levels (0.01, 0.001, and 0.0001) and five levels (0, 25, 50, 75, and 100), respectively. The X-axis represents these parameter combinations, while the Y-axis represents the following metrics: Precision (calculated by dividing the number of true positive genes by the total number of detected genes), Recall (calculated by dividing the number of true positive genes by the total number of positive genes), Alignment rate (percentage of reads aligned to the ChocoPhlAn3 pangenome database), and Running Time (measured in minutes). The red line represents the combination of parameters used in subsequent analysis.

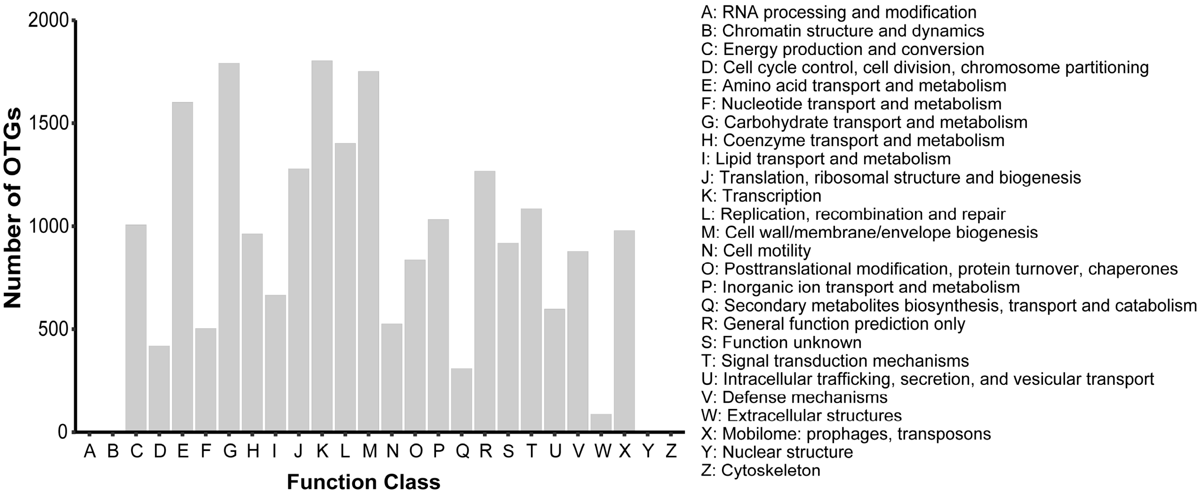

Figure S2. Number of OTGs involved in different COG classifications in CRC groups. The x-axis represents the names of 26 groups, and the y-axis corresponds to the number of OTGs in each group.

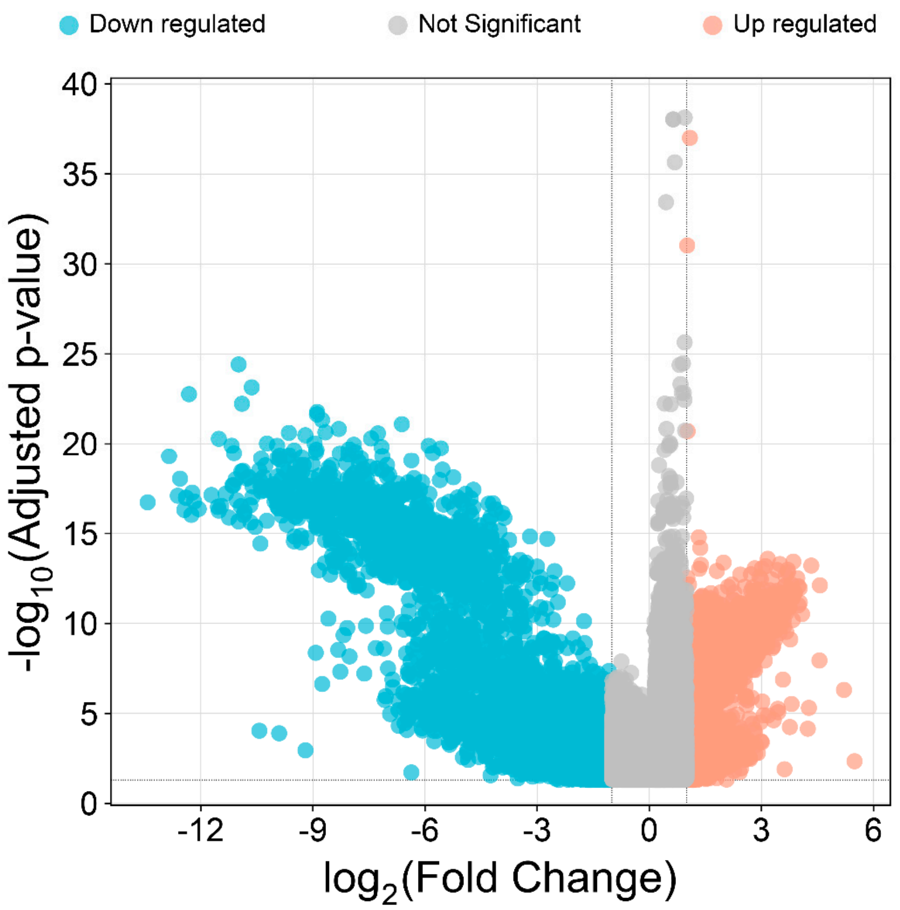

Figure S3. Volcano plot of oral translocation genes between the healthy control group and the colorectal cancer group. The plot presents the log2(Fold Change) (x-axis) against the -log10(adjusted p-value) (y-axis). Genes with log2FC > 2 and adjusted p-value < 0.05 were classified as upregulated (red), while genes with log2FC < -2 and adjusted p-value < 0.05 were classified as downregulated (blue). Non-significant genes are displayed in grey. Vertical dashed lines indicate the log2FC thresholds, and the horizontal dashed line marks the significance threshold.

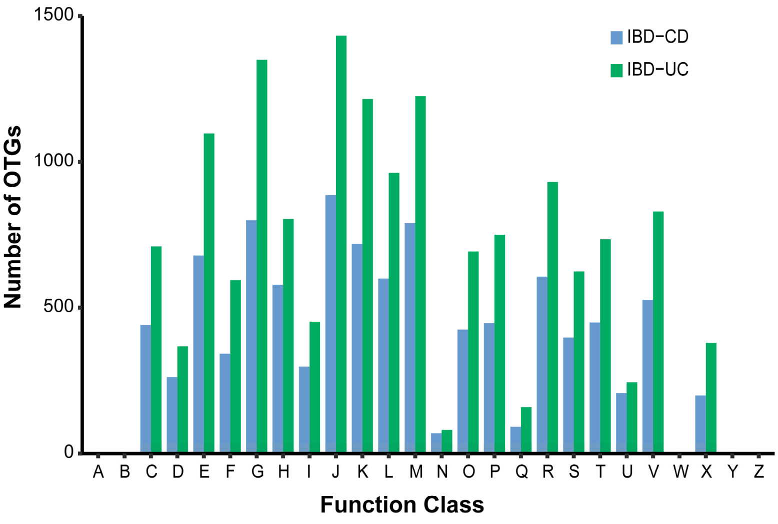

Figure S4. Number of OTGs involved in different COG classifications in CD and UC groups. The x-axis represents the names of 26 groups, and the y-axis corresponds to the number of OTGs in each group.

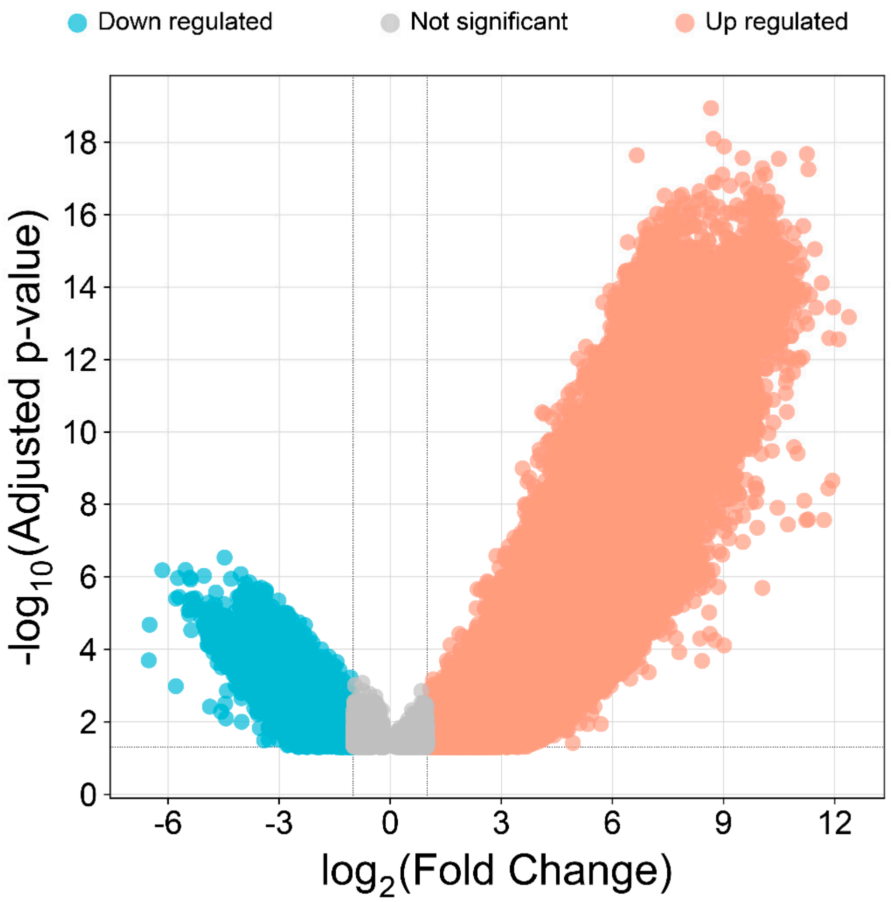

Figure S5. Volcano plot of oral translocation genes between the healthy control group and the Crohn's disease group. The plot presents the log2(Fold Change) (x-axis) against the -log10(adjusted p-value) (y-axis). Genes with log2FC > 2 and adjusted p-value < 0.05 were classified as upregulated (red), while genes with log2FC < -2 and adjusted p-value < 0.05 were classified as downregulated (blue). Non-significant genes are displayed in grey. Vertical dashed lines indicate the log2FC thresholds, and the horizontal dashed line marks the significance threshold.

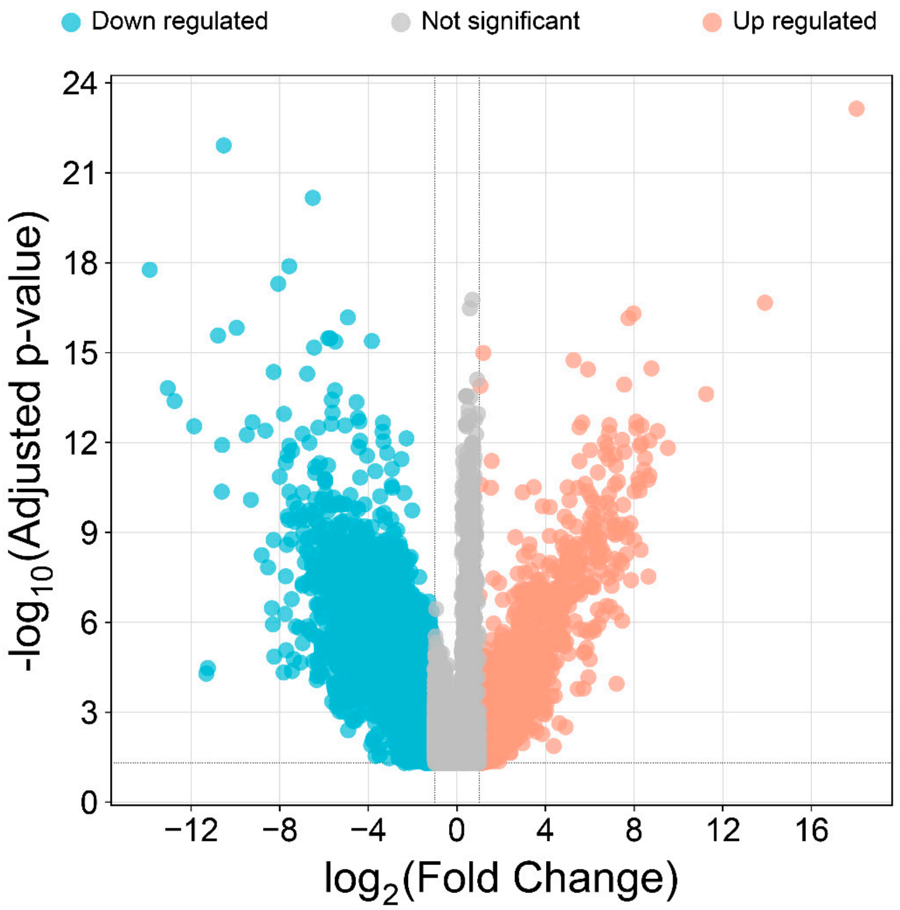

Figure S6. Volcano plot of oral translocation genes between the healthy control group and the ulcerative colitis group. The plot presents the log2(Fold Change) (x-axis) against the -log10(adjusted p-value) (y-axis). Genes with log2FC > 2 and adjusted p-value < 0.05 were classified as upregulated (red), while genes with log2FC < -2 and adjusted p-value < 0.05 were classified as downregulated (blue). Non-significant genes are displayed in grey. Vertical dashed lines indicate the log2FC thresholds, and the horizontal dashed line marks the significance threshold.
